# Exposure Assessment of Heavy Metals and Microplastic-like Particles from Consumption of Bivalves

**DOI:** 10.3390/foods12163018

**Published:** 2023-08-11

**Authors:** Pharrunrat Tanaviyutpakdee, Weeraya Karnpanit

**Affiliations:** 1 Institute of Nutrition, Mahidol University, 999, Salaya, Phutthamonthon, Nakhon Pathom 73170, Thailand; 2 School of Science, Western Sydney University, Locked Bag 1797, Penrith, NSW 2751, Australia

**Keywords:** lead, cadmium, microplastic-like particle, bivalve, exposure

## Abstract

The aim of this study was to determine the contamination of lead (Pb), cadmium (Cd) and microplastic (MP)-like particles in bivalves and estimate the exposure of the Thai population to these contaminants due to bivalve consumption. Clams, mussels and cockles were purchased from five wholesale seafood markets located on the upper Gulf of Thailand during the period 2017–2019. Determinations of Cd and Pb in the bivalves were conducted using a graphite furnace atomic absorption spectrometer (GFAAS). Visualization was conducted using a stereomicroscope to investigate the morphology and content of MP-like particles in the bivalve samples. The average Pb contents in clams, mussels and cockles were 112, 64 and 151 µg/kg wet wt., respectively. The average Cd contents were 126, 107 and 457 µg/kg wet wt. for clams, mussels and cockles, respectively. The average number of MP-like particles in bivalve samples varied from not detected to 1.2 items/g wet wt. and not detected to 4.3 items/individual. The exposure to Pb, Cd and MP-like particles due to bivalve consumption varied between 0.005 and 0.29 µg/kg bw/day, 0.017 and 28.9 µg/kg bw/month and 0.015 and 27.5 items/person/day, respectively. There was no potential health risk of exposure to Pb and Cd due to bivalve consumption in any age group. However, a high consumption of cockles with high Cd levels (the worst-case scenario) in children may be of concern.

## 1. Introduction

The global consumption of bivalves has increased with growing interest in their nutritional benefits [1]. More than 15 million tons of bivalves are produced annually, accounting for 14% of the total marine production worldwide [2]. Asian countries account for over 85% of global bivalve production. Most of the bivalve production (approximately 90%) comes from aquaculture [2]. According to the FAO Global Fishery and Aquaculture Statistics database, bivalve species can be classified into four major groups: clams, cockles, mussels and oysters [2]. Bivalves are considered to be highly nutrient-dense and sustainable to produce [1,3]. Bivalves are good sources of protein and some micronutrients such as vitamin B_12_, iron and zinc [3]. However, bivalves are likely to be contaminated by toxic compounds in seawater and sediment because of their filter-feeding activity [1].

Heavy metals are ubiquitous and persistent in the environment. Heavy metal toxicity is a major environmental health problem due to bioaccumulation through the food chain [4]. Because of industrial and agricultural development, heavy metal pollution has been a serious health concern for several decades. Lead (Pb) and cadmium (Cd) are two of the most common heavy metals that frequently coexist in the environment [5]. Chronic exposure to Pb poses adverse health effects in respect to various systems, including the blood circulatory, cardiovascular, endocrine, gastrointestinal, immune, nervous, renal and reproductive systems [4,6]. The International Agency for Research on Cancer (IARC) has classified Pb in group 2A, i.e., probably carcinogenic to humans [7]. Consistent evidence of associations between impaired neurodevelopment and blood Pb concentration in children has been reported [8]. Lead poisoning causes cerebral palsy in children, and affects their level of intelligence [9]. The adverse effects of Pb exposure on the cardiovascular system, associated with an increasing systolic blood pressure in adults, have been clearly demonstrated [8]. Consequently, developmental neurotoxicity measured by reference to decreased IQ scores in children, and cardiovascular effects measured by reference to increased systolic blood pressure in adults, have been selected as the most sensitive end-points for dose–response assessment [8]. The previous health-based guidance value (HBGV) of Pb as a provisional tolerable weekly intake (PTWI) of 25 μg/kg bw was withdrawn in 2011 because it could no longer be considered health protective [8]. According to the effect of Pb on IQ reduction in children and the causing of kidney damage in adults, benchmark dose levels (BMDLs) of BMDL_01_ of 0.50 µg/kg bw/day and BMDL_10_ of 0.63 µg/kg bw/day have been established for children and adults, respectively [10].

Long-term exposure to Cd affects various toxicological end-points in experimental animals, including reproductive toxicity, neurotoxicity and carcinogenicity. However, the kidneys are the most sensitive organs in humans and other mammals exposed to Cd [8]. An association between Cd concentration in the kidneys and morphological and/or functional changes in the kidneys have been reported. The IARC has classified Cd as a group 1 carcinogen, with sufficient evidence for lung cancer in humans from many epidemiological studies on occupational exposure to Cd via inhalation [4,8]. Due to the long half-life of Cd, a provisional tolerable monthly intake (PTMI) of 25 μg/kg bw/month has been established. This HBGV was derived from a critical concentration of Cd in the kidneys causing an increase in β_2_-microglobulin (β2MG) concentration in urine, and a toxicokinetic model of dietary exposure to Cd and its bioaccumulation in the kidneys [8,11].

Microplastic (MP) pollution in the environment has become a global concern [12]. Microplastic is defined as plastic material with sizes of less than 5000 µm [13]. Microplastics can be transferred from the environment to humans through the food chain, which may increase the potential health risks to humans [12]. The toxicity of MPsis likely dependent on size, associated chemicals and dose. Adverse health effects due to MPs may occur from polymers, additive chemicals and other chemicals in the environment, which can be adsorbed to MP particles, including persistent organic pollutants (POPs) [14]. However, studies on the toxicity of MPs in mammals are limited. A previous study investigated the toxicity of polystyrene microplastic (diameters of 5 and 20 µm) exposure in mice, showing that MPs were accumulated in the liver, kidney and gut. In addition, the study in mice indicated that MP exposure caused various toxic responses, such as diminished energy and lipid metabolism, increased oxidative stress and biomarkers of neurotoxicity [15]. To date, there is no health-based guidance value established for MPs. Most of the previous animal studies on MP exposure examined the toxicity of single polymers such as polyamide, polyethylene and polystyrene, making it impossible to extrapolate the data from animals to human exposure scenarios involving multiple polymers [16]. 

Bivalves are used as bio-indicators for marine pollution, including heavy metals and MPs, because they are filter feeders accumulating various contaminants [17]. After deshelling a bivalve, people usually consume the whole organism, including the gut, making it a high-risk food for exposure to heavy metals and MPs. The aim of this study was to determine the levels of Pb, Cd and MP-like particles in bivalves collected from Thailand. An exposure assessment of the contaminants was performed to characterize the health risk to the Thai population due to the consumption of bivalves in order to monitor contaminant levels in high-risk foods and provide consumption advice.

## 2. Materials and Methods

### 2.1. Reagents

Lead and cadmium standard solutions (TraceCERT^®^ 1000 mg/L) for AAS were purchased from Supelco (Bellefonte, PA, USA). Concentrated nitric acid (65%), ammonium solution (25%) and diammonium hydrogen citrate were purchased from Merck (Darmstadt, Germany). Chloroform was purchased from LabScan (Bangkok, Thailand). Ammonium-1-pyrrolidine dithiocarbamate, polyethylene, polyethylene terephthalate, polypropylene, polystyrene and polyvinyl chloride were purchased from Sigma-Aldrich (St. Louis, MO, USA). 

### 2.2. Bivalve Samples

**Figure 1:**
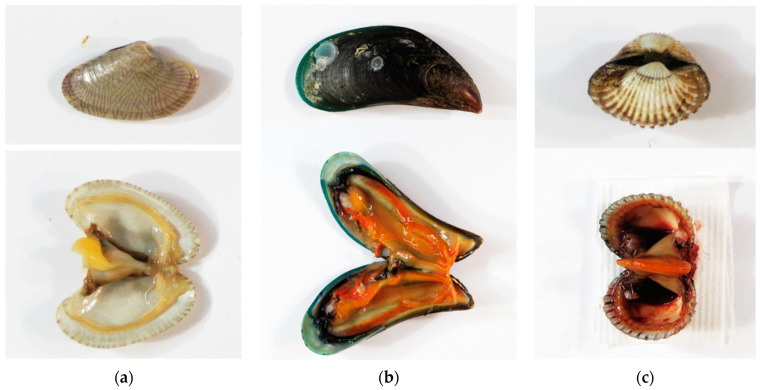
Photographs of whole mollusk and the edible part inside of individual species: (**a**) clam (*Paphia undulata* (Born, 1778)); (**b**) mussel (*Perna viridis* (Linnaeus, 1758)); (**c**) cockle (*Anadara granosa* (Linnaeus, 1758)).

Undulated surf clam (*Paphia undulata* (Born, 1778)), green mussel (*Perna viridis* (Linnaeus, 1758)) and bloody cockle (*Anadara granosa* (Linnaeus, 1758)) were the three types of bivalve mollusks selected for the present study (Figure 1). The common short-form names of these species, i.e., clam, mussel and cockle, are used throughout this article. The bivalve mollusks were purchased from five large wholesale seafood markets in Samut Sakhon and Samut Songkhram provinces located in the upper Gulf of Thailand. Approximately 1–2 kg of samples were collected randomly from three shops in each market. The samples were immediately stored in an ice box and transported to the laboratory for sample preparation. Bivalve samples were collected in three seasons in three different years; namely the rainy season (June–July 2017), winter (December 2018) and summer (May 2019). The bivalve samples were prepared by washing with tap water and deionized water. Shells were removed and the weights of whole mollusks (shell and meat) and meat were recorded. Only edible parts of individual bivalve mollusk species were homogenized using a blender and stored in an air-tight container in a freezer at −20 °C until further analysis.

### 2.3. Moisture Content Determination

The moisture contents of the homogeneous bivalve samples were determined based on the AOAC 950.46 method [18]. Approximately 2 g of the sample was weighed into a dish. The dish was then evaporated until dry in a hot-air oven at 102 °C. The dish was then cooled in a desiccator and the process was repeated until a constant weight was reached. Moisture contents were used for conversion of a wet weight basis to a dry weight basis.

### 2.4. Analysis of Pb and Cd Levels in Bivalve Samples

Sample digestions for Cd and Pb determination were conducted according to Sadiq et al. [19] with some modifications. Approximately 1 g of the sample was weighed into a Teflon jar. The Teflon jar was heated for at least 9 h at a temperature of 110 °C until complete digestion. Ammonium-1-pyrrolodinedithiocarbamate (APDC) solution as a metal chelator was added to the digestate to form complexes with heavy metals. Nitric acid (3%) solution was used to dissolve the heavy metals for further analysis using a graphite furnace atomic absorption spectrometer (model PinAAcle 900Z, PerkinElmer, CT, USA). The limits of detection (LOD) for the determination of Pb and Cd were 4 and 3 μg/kg, respectively. The limits of quantitation (LOQ) for Pb and Cd determination were 13 and 10 μg/kg, respectively. 

Quality control of the Pb and Cd analyses related to the accuracy, precision and linearity of the working range were performed in every batch. Accuracy was determined via the recovery rate of spiked samples. Spiked samples were created using the spiking mixed standards of Pb and Cd at concentrations of 200 and 20 µg/L, respectively. The recovery rates of Pb and Cd were in the ranges 81–108% and 85–110%, respectively, which were within an acceptable range according to AOAC 2019 (80–110%). Precision was estimated via triplicate analysis. Triplicate samples were analyzed for Pb and Cd contents, and the relative standard deviations (RSDs) were calculated. The relative standard deviations of the Pb and Cd analyses ranged between 2.8 and 8.7% and 4.1 and 9.6%, respectively, which were not greater than 15% of the acceptable criteria for the analysis value at 100 µg/kg. The working ranges of the calibration curves for Pb and Cd were 10–100 μg/L and 2–8 μg/L, respectively. The coefficients of determination (r^2^) of the calibration curves were greater than 0.99, indicating that the calibration curves of the Pb and Cd analyses in this study fitted with the linear regression line. All glassware were cleaned by soaking in 20% nitric acid overnight, then rinsed with deionized water and dried in a hot-air oven.

### 2.5. Determination of MP-like Particle Contents in Bivalve Samples

Determination of the MP contents in bivalve samples was performed according the method of Li et al. [20] with some modifications. Approximately 5 g of cockle or 10 g of clam or mussel samples was weighed into a 250-mL Erlenmeyer flask. Thirty (30) mL of 30% hydrogen peroxide solution was added to each individual flask. Each flask was covered and placed at room temperature for 1–2 weeks until the soft tissue of the bivalve was completely digested. After digestion, the digestate was centrifuged at 4000 rpm for 10 min and filtered through filter paper (WhatmanTM grade 5, 70 mm diameter, 2.5 µm pore size) coupled with a vacuum pump. The filter paper was dried in a hot-air oven at 40 °C for one hour. The filter paper was kept in a Petri dish with a cover until microscopic detection. To detect MP-like particles, the filter paper was examined under a stereomicroscope (model 305, Carl Zeiss, Oberkochen, Germany) at 20× to 40× magnification with ZEN 3.0 Blue edition software. Characterization of MP-like particles was conducted following the method of Hidalgo-Ruz et al. [21]. Important characteristics of MP-like particles included no cellular or organic structures visible and an equal thickness throughout the entire length of fiber-shaped items. 

To prevent the contamination of MPs during the experiment, a procedural blank was conducted and laboratory benches were cleaned with 70% ethanol. Nitrile and powder-free gloves were worn throughout the experiment. To determine the recovery rate of MP particles, spiking of the fine particles of polymers with known weights was conducted, following the method of Karami et al. [22] with some modifications. Five types of polymers (namely, polyethylene (PE), polyethylene terephthalate (PET), polypropylene (PP), polystyrene (PS) and polyvinyl chloride (PVC)) were used for the recovery test. Approximately 0.03 g of each polymer (PE, PP, PS and PVC) except for PET (3 pieces of pellets ca. 0.07 g) was weighed into Erlenmeyer flasks, digested with 30 mL of H_2_O_2_, centrifuged, filtered through filter paper and dried in a hot-air oven. The weight of the spiked polymer after digestion was recorded. The percentage of recovery was calculated from the weight of the spiked polymer before and after the MP isolation process. None of the MP-like particles were detected on the filter papers of procedural blank flasks under the stereomicroscope. The recovery rate of spiked polymers ranged from 91.2 to 95.7%. The high recovery rate indicates insignificant loss and degradation of plastic particles during the experiment.

### 2.6. Bivalve Mollusk Consumption Data for the Thai Population

Data on bivalve mollusk consumption for the Thai population were obtained from the national consumption survey of Thailand [23]. According to that survey, clams, mussels and cockles were the top three bivalves consumed. Bivalve consumption data were used to estimate the Thai population’s exposure to Pb, Cd and MP-like particles. The bivalve consumption data were presented in terms of cooked food; therefore, the levels of Pb, Cd and MP-like particles in raw bivalves were converted to the levels of cooked bivalves. Cooking factors for each bivalve from the previous study of Banjong et al. [24] were applied for the calculation. Cooking factors of 1.05, 1.65 and 1.62 were applied for clams, mussels and cockles, respectively.

### 2.7. Assessment of Exposure to Pb, Cd and MP-like Particles Due to Bivalve Consumption

A deterministic risk assessment of the Thai population’s exposure to Pb, Cd and MP-like particles due to bivalve consumption was conducted according to the guidelines of the WHO [25]. Average and 97.5 percentile (PCTL) consumption of bivalves (per capita), and average and 97.5 PCTL of contaminants, were considered for four exposure scenarios:

(1) Average exposure (average consumption x average concentration);
(2) High contaminant level exposure (average consumption × 97.5 PCTL concentration);
(3) High consumption exposure (97.5 PCTL consumption × average concentration);
(4) Worst-case exposure (97.5 PCTL consumption × 97.5 PCTL concentration).

For exposure to heavy metals, only the upper-bound scenario was performed by replacing heavy metal levels below the LOD with the LOD value and the levels below the LOQ with the LOQ value. Exposure was calculated using the following equations:

For Pb:

Exposure (µg/kg bw/day) = Consumption × Concentration(1)

For Cd:

Exposure (µg/kg bw/month) = Consumption × Concentration(2)

For MP-like particles:

Exposure (item/person/day) = Consumption × Number of MPs(3)

From the national food consumption data of Thailand, the food consumption of consumers was calculated from a single day of consumption for acute exposure assessment to chemicals. Therefore, data in respect to bivalve consumption as consumers only were not applied for the exposure assessment in this study.

### 2.8. Risk Characterization of Exposure to Pb, Cd and MP-like Particles Due to Bivalve Consumption

The risk of Pb exposure was characterized by comparing Pb exposure with a BMDL_01_ of 0.5 µg/kg bw/day for neurodevelopmental effects in children, and a BMDL_10_ of 0.63 µg/kg bw/day for renal toxicity in adults. The BMDL of Pb was estimated using the results of human studies; therefore, a margin of exposure (MOE) greater than 1 is considered a very low health risk [10]. The risk characterization of Cd exposure due to bivalve consumption was estimated by comparing the Cd exposure with a PTMI of 25 µg/kg bw/month. 

The risk characterization of Pb exposure due to bivalve consumption was calculated as the MOE using the following equation:

MOE = BMDL/Exposure to Pb(4)

where MOE < 1 indicates that there might be adverse health effects.

The risk characterization of Cd exposure due to bivalve consumption was calculated as a hazard quotient using the following equation:

Hazard Quotient (HQ) = Exposure to Cd/PTMI(5)

where HQ > 1 indicates that there might be adverse health effects.

To date, there is no HBGV established for microplastics; therefore, the risk characterization of MP-like particles could not be evaluated. 

### 2.9. Statistical Analysis

Statistical tests were conducted using the SPSS Statistical Analysis Software Program version 19. Lead, cadmium and MP-like particle levels in bivalve samples are presented as mean and standard deviation. For a parametric distribution, one-way analysis of variance (ANOVA) and Tukey’s test were used to examine statistical significance. For a non-parametric distribution, the Kruskal–Wallis test and Mann–Whitney U-test were used to examine statistical significance. A probability level of *p* < 0.05 was considered statistically significant.

## 3. Results

### 3.1. Lead and Cadmium Contents in Bivalves

Lead and cadmium contents in bivalves are presented as mg/kg wet weight and mg/kg dry weight (Table 1). As shown by the results, almost all bivalve samples contained Pb and Cd. Only 4.2% (1/24 samples), 7.4% (2/27 samples) and 3.7% (1/27 samples) of clam, mussel and cockle samples, respectively, contained Pb contents at lower levels than the detection limit. For Cd contamination, all clam and cockle samples contained Cd at levels above the LOQ. Only one mussel sample had a Cd level lower than the LOD. 

There were significant differences in Pb and Cd contents among the bivalve species (Table 1). The highest mean contents of Pb and Cd were found in the cockle samples. Clams had significantly higher Pb levels (wet weight basis) than the mussel samples. However, there was no significant difference between the Pb concentrations (dry weight basis) of clam and mussel samples. There was also no significant difference between the Cd concentrations of clam and mussel samples (wet weight and dry weight basis).

Considering seasonal and annual variations, there were significant differences in the Pb contents of individual bivalve species collected in different seasons and years (Table 1). For Pb contamination, the highest mean Pb content was found in the three bivalve species collected in winter. For Cd contamination in clams, the highest average Cd content was found in clam samples collected in winter, while there was no significant difference between the contents in the summer and rainy seasons. For the contamination of Cd in mussels, there was no significant difference between samples collected in different seasons and years. For the contamination of Cd in cockles, there was no significant difference between samples collected in winter and summer. The lowest Cd concentrations (on a wet weight and dry weight bases) for cockles were found in the samples collected in the rainy season.

### 3.2. MP-like Particles Detected in Bivalves

Microplastic-like particle abundances in the three types of bivalves collected in the present study are shown in Table 2. The detection rate of MP-like particles in bivalve samples was 30.3% (23/76 samples). Microplastic-like particles were found in clam, mussel and cockle samples at detection rates of 31.8% (7/22 samples), 29.6 (8/27 samples) and 29.6% (8/27 samples), respectively. In total, 49 MP-like particles were found in three species of bivalve samples, with 14, 19 and 16 items detected in clams, mussels and cockles, respectively. The total numbers of MP-like particles varied from not detected to 1.2 items/g for the edible parts of the bivalve. The average contents of MP-like particles found in clam, mussel and cockle samples were 0.06, 0.07 and 0.12 items/g, respectively. It was found that the average amounts of MP-like particles in clam, mussel and cockle samples were 0.14, 0.60 and 0.38 items/individual, respectively. 

There were significant differences in MP-like particle levels among bivalve species. Regarding the number of MP-like particle items per gram of sample, the highest number of MP-like particles was found in cockles. There was no significant difference between the numbers of MP-like particles found in clams and mussels. Considering seasonal and annual variations, there were significant differences in the MP-like particle numbers of individual bivalve species collected in different seasons and years. For MP-like particle accumulation in clams, the highest number of MP-like particles was found in the clam samples collected in winter, followed by those from summer and the rainy season. For MP-like particle contamination in mussels, the highest number of MPs was found in mussel samples collected in the rainy season, followed by those from summer and winter. For MP-like particle accumulation in cockles, there was no significant difference in MP-like particle numbers in cockle samples collected in the rainy and summer seasons, while the lowest number of MPs was found in cockles collected in the winter season.

**Figure 2:**
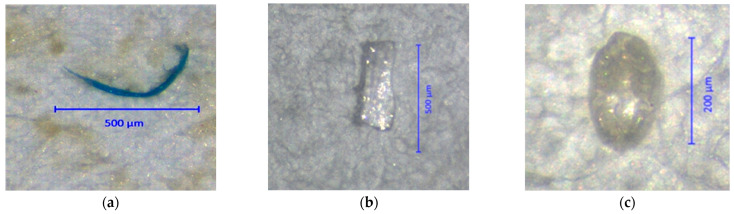
Stereomicroscopic images of MP-like particles detected in bivalve samples: (**a**) fiber; (**b**) fragment; (**c**) pellet. The filter papers were examined under a stereomicroscope at 40× magnification.

**Figure 3:**
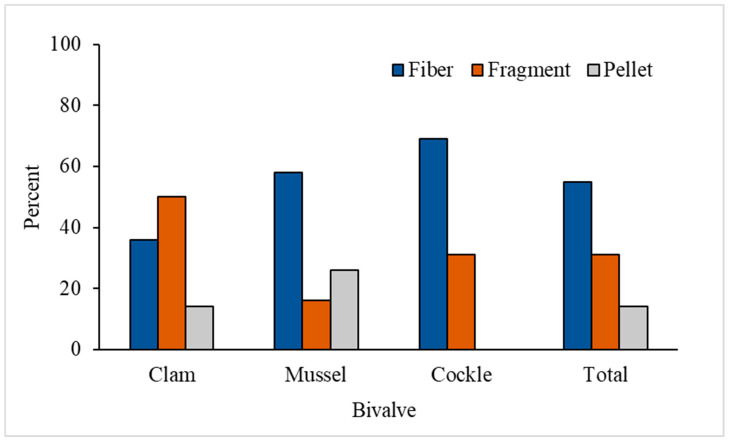
Shape distribution of MP-like particles detected in bivalves.

Various shapes of MP-like particles classified as fibers, fragments and pellets were detected in the bivalve samples (Figure 2). The shape distribution of MP-like particles detected in bivalves are shown in Figure 3. Among the 49 items of MP-like particles found in the three bivalve species, 55.1% (27/49 items) were fibers, 30.6% (15/49 items) were fragments and 14.3% (7/49 items) were pellets. It was found that the 14 MP-like particles found in clam samples consisted of 5 fibers (35.7%), 7 fragments (50.0%) and 2 pellets (14.3%). For mussels, 19 MP-like particles were detected, in which there were 11 fibers (57.9%), 3 fragments (15.8%) and 5 pellets (26.3%). For cockles, 16 MP-like particles were detected, in which there were 11 fibers (68.8%) and 5 fragments (31.2%). Different colors of MP-like particles were found in fibers, including orange, blue and pink. Most pellets were colorless; however, three pellet colors were observed, i.e., orange, brown and purple. Most of the fragments were colorless except for one item, which was orange. 

**Figure 4:**
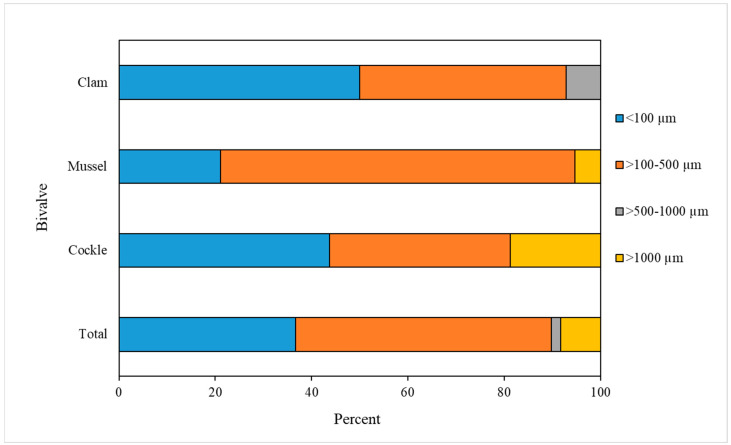
Size distribution of MP-like particles detected in bivalves.

The sizes of MP-like particles detected were approximately in the range 20–1500 µm. Figure 4 shows the size distribution of MP-like particles found in the bivalve samples. The sizes of the MP-like particles found were classified into four groups: ≤100 µm, >100–500 µm, >500–1000 µm and >1000 µm. The most abundant size range of MP-like particles was >100–500 µm; this size range was found for 26 items of all the MP-like particles detected (53.1%), followed by the ≤100 µm size (18/49 items, 36.7%), and the >1000 µm size (4/49 items, 8.2%). The least abundant size range of MP-like particles detected was >500–1000 µm (1/49 items, 2.0%). However, because of the inability to extend fiber items for exact measurements, the sizes of MP-like particles, especially fibers, are approximate values.

### 3.3. Exposure Assessment and Risk Characterization of Exposure to Pb and Cd from Bivalve Consumption

Results in respect to the exposure to and MOEs of Pb due to bivalve consumption by the Thai population are shown in Table 3. From the average exposure scenario, the highest lead exposures were found to be due to the consumption of cockles in all age groups (0.0014–0.014 µg/kg bw/day). All MOEs were much greater than 1 (36–450), indicating very low risk. From the worst-case exposure scenario, the highest Pb exposures were also found to be due to the consumption of cockles (0.022–0.288 µg/kg bw/day). All MOEs were greater than 1 (3–29), indicating low risk of concern.

Exposure to Cd and HQ due to bivalve consumption by the Thai population is shown in Table 4. From the average exposure scenario, the highest Cd exposures were found to be due to the consumption of cockles in all age groups (0.13–1.25 µg/kg bw/month). All HQs were much lower than 1 (0.005–0.50), indicating very low risk. From the worst-case exposure scenario, the highest Cd exposures were also found to be due to the consumption of cockles (2.16–28.86 µg/kg bw/month). It was found that the HQ of Cd exposure in children (6–12.9 years) slightly exceeded 1, indicating a potential health risk.

### 3.4. Assessment of Exposure to MP-like Particles Due to Bivalve Consumption

Results for the exposure assessment of MP-like particles due to bivalve consumption by the Thai population are shown in Table 5. From the average exposure scenario, the highest MP-like particle exposures were found to be due to the consumption of cockles in all age groups (0.06–0.40 item/person/day). From the worst-case exposure scenario, the highest MP-like particle exposures were also found to be due to the consumption of cockles (3.44–27.5 item/person/day).

## 4. Discussion

### 4.1. Lead and Cadmium Contents in Bivalves

In this study, it was found that cockles were likely to accumulate higher Pb and Cd contents than the other two bivalves. This could be due to cockles burying themselves in mud beaches of 1–12 inches in estuary and coastal areas [26]. Estuary and coastal areas might be polluted with waste water and chemical substances from industries and communities. Mussel farming involves mussels settling at a depth of 4–6 meters in the sea or being raised on a rope line at a deep-water level off the coast. Clams live in the depths of the sea up to 8 meters deep; these mollusks also bury themselves up to 20 centimeters under the mud [27].

A previous study reported that among seafoods, mollusks had a high accumulation rate of heavy metals [28]. This is because mollusks consume foods by filtering seawater through their bodies. Therefore, they are able to filter various heavy metals and accumulate them in their tissues. For this reason, bivalves are used as indicators for monitoring heavy metal pollution in the environment. Previous research on heavy metal contamination in marine animals concluded that the accumulation of heavy metals in marine animals depends on their habitats, dietary habits, age, size and duration of exposure to heavy metals [29].

According to the Notification of the Ministry of Public Health (No 414) B.E. 2563, the maximum level (ML) of Cd in bivalves has been set at 2 mg/kg, and the ML of Pb in foods has been established at 1 mg/kg [30]. The Codex Alimentarius Commission has also established a ML of Cd at 2 mg/kg; however, the ML of Pb has not been determined in bivalves by the commission [31]. From Regulation (EC) No. 1881/2006 of the European Union, the MLs of Cd and Pb in bivalves have been set at 1 and 1.5 mg/kg, respectively [32]. In this study, none of the bivalve samples had Cd and Pb contents exceeding the maximum levels established by the Ministry of Public Health of Thailand, the Codex Alimentarius Commission or the European Union.

The average Pb and Cd levels (wet weight basis) detected in bivalves in the present study were lower than those in some previous studies. A previous study reported average levels of Cd in mussels (0.797 mg/kg) and clams (0.251 mg/kg), and average Pb level in clams (0.141 mg/kg) collected from coastal areas of Southeast China [33]. In a previous study in Vietnam, mussels and cockles collected from local markets were contaminated with high levels of Pb at 0.71 and 0.70 mg/kg, respectively [34]. High average Cd and Pb levels of 0.67 and 0.94 mg/kg, respectively, were reported in clams obtained from the East Java coast of Indonesia [35].

Similar to the present study, higher average Cd levels in cockles compared to mussels collected in Thailand have been reported [36]. Seasonal variations significantly affect heavy metal accumulation in bivalve species. A previous study reported the highest contents of Cd and Pb in mussels and clams collected in the winter [37]. Similar to our study, higher Cd and Pb levels in cockles collected in the winter season were reported compared to those of the rainy season [38].

### 4.2. Microplastic-like Particles in Bivalves

Hydrogen peroxide solution (30% *v*/*v*) was used to digest the organic matter of bivalve samples in this study. From previous studies, it was reported that H_2_O_2_ is the most appropriate solution for MP detection because it does not destroy polymers [39,40]. Some problems occurred during the examination of MPs on the filter papers because of the ambiguous characteristics of non-plastic and plastic items, especially the fragment-shaped items. However, this could be overcome by magnifying the suspected objects under the stereomicroscope. Biotic items contain cell structures, while these structures cannot be detected in abiotic objects [21]. The limitations of this study also include the inability to verify the types of polymers detected. The samples need to be further analyzed to confirm the polymer types using advanced instruments such as a Fourier transform infrared spectrometer or a Raman spectrometer.

The abundances of MPs in the bivalves of this study were similar to those in some previous studies. A previous study reported the abundance of MPs in oysters collected from 17 sites along the coastline of China. The average abundance of MPs in oyster samples was 0.62 items/g wet weight or 2.93 items/individual [41]. Microplastic amounts found in four species of bivalves (oyster, mussel, clam and scallop) sold in fishery markets of South Korea were reported. The mean levels of MPs in four bivalve species samples were 0.15 ± 0.20 item/g wet wt. and 0.97 ± 0.74 items/individual [42]. However, the abundances of MPs in bivalves in this study were lower than those of some previous studies. The average contents of MPs detected in mussels from different commercial and natural sources were in the range 4.4–11.4 items/g wet wt. and 3–12.4 items/mussel, respectively [43]. A previous study found high levels of MP contamination in nine species of bivalves collected from a market in China, varying from 4.3 to 57.2 items/individual [20]. These could be due to the contamination levels of MPs in the environment where the mollusks live.

Similar to previous studies, the most abundant type of MP in the bivalve samples of this study was fibers. A previous study reported that all MPs detected in farmed and natural mussels were fibers with lengths of 750–6000 μm [43]. Another study reported that fibers were the most commonly found MPs in bivalves and consisted of more than half of the total MPs in each of the eight species [20]. The fact that fibers are the most abundant MPs detected in bivalves could probably be due to the fact that they are the most prevalent type of MPs found in the environment [44]. In addition, the morphology of these filaments allows them to be easily accumulated in bivalve species. 

### 4.3. Exposure Assessment and Risk Characterization of Pb and Cd from Bivalve Consumption

From the results of all Cd and Pb exposure scenarios, Cd and Pb exposure due to bivalve consumption in all age groups did not exceed the PTMI and BMDL for Cd and Pb, respectively. However, from the worst-case scenario, Cd exposure due to cockle consumption in children exceeded the HBGV, which may pose adverse health effects. A previous study reported that exposure to Cd and Pb from bivalve consumption in Vietnamese people did not exceed the PTMI, indicating no health risks of concern [34]. A previous study reported average estimated daily intakes of Cd (11.01 μg/kg bw/month) and Pb (0.087 μg/kg bw/day) due to the consumption of six bivalve species collected from local markets in Southeast China, which were much higher than the average exposure to Cd and Pb due to the consumption of the three bivalve species in this study. The authors also reported a potential cancer risk from Cd exposure due to bivalve consumption from the exposure scenario using the 97.5th percentile concentration of Cd [33]. A high cancer risk due to Cd intake from the consumption of clams with high Cd contents collected from Indonesia was also reported [35]. 

In our study, we collected bivalve samples from seafood markets to represent the consumption of the general population and characterize the health risk to Thai people. However, bivalve samples collected from industrial areas contain high levels of heavy metals and may pose a health risk to consumers. A previous study reported high Cd contamination in scallops from the coast of Map Ta Phut Industrial Estate, Rayong province in Thailand, and observed that consumers were at risk of exposure to Cd due to consuming scallops caught from that particular coastal area [45]. 

### 4.4. Assessment of Exposure to MP-like Particles Due to Bivalve Consumption

Microplastic-like particle exposure due to bivalve consumption by the Thai population calculated for the average scenario and worst-case scenario varied between 0.015 and 27.5 items/person/day, which was equal to the range 5.48–10,038 items/person/year. The considerably high MP exposure shown in this study could be due to the worst-case exposure scenario being examined. This scenario might be an over-estimation and may rarely occur; however, it could be applied as a screening approach for exposure assessment. Dietary exposure to MPs via shellfish intake varies considerably among different countries. An average intake of 212 items/person annually via the consumption of four species of bivalves (oysters, mussels, clams and scallops) has been reported for the South Korean population [42], while MP intake via shellfish consumption in European people was in the range 1800–11,000 items/person/year [46].

Microplastics with sizes smaller than 150 µm may translocate to the lymph and circulatory systems; however, less than 0.3% of the ingested MPs might be absorbed [47]. It has been reported that MPs with a size of <20 µm could pass into certain organs. At present, the potential adverse health effects of MPs have been reported, including increased inflammatory response, immunotoxicity and gut microbiome disruption [47,48]. A reduction in MP amounts by the depuration of bivalves in clean water prior to sale to consumers could reduce the amount of MP contamination [49]. A previous study reported that cooking may reduce MP contamination from bivalves. Cooking mussels for 2 min in boiling water could decrease the MP contents by 14% compared to raw mussels [43]. Microplastics have also been detected in cooking water and are characterized by a smaller size than in raw mussels. The results indicate that some MP particles might leach into cooking water; however, using some other cooking methods such as grilling and baking might not reduce the MP contamination in bivalves [43]. 

## 5. Conclusions

Cockles are more likely to accumulate Cd and Pb at higher levels than those of clams and mussels. The highest number of MP-like particles (as items per gram of sample) was also found in cockles. Among the three bivalves studied, it is likely that cockles are the major contributor of Pb and Cd exposure in the Thai population. Exposure to Cd and Pb due to the consumption of bivalves by the Thai population is not of concern, since none of the estimated exposure scenarios exceeded the relevant safety levels. However, the Cd intake due to cockle consumption in children estimated from the worst-case scenario suggested a potential health risk. Data on Cd, Pb and MP contamination in bivalves, and exposure to these contaminants due to the consumption of bivalves, could be disseminated to food safety authorities to establish food regulations and provide consumption advice.

## Figures and Tables

**Table 1:** Lead and cadmium contents of bivalve mollusks.

Type of Bivalve	Collection Year	Heavy Metal Contents			
		Pb (mg/kg Wet wt.)	Cd (mg/kg Wet wt.)	Pb (mg/kg Dry wt.)	Cd (mg/kg Dry wt.)
Clam	Rainy, 2017	0.078 ± 0.037 ^c^	0.103 ± 0.064 ^b^	0.497 ± 0.239 ^c^	0.630 ± 0.403 ^b^
	Winter, 2018	0.219 ± 0.062 ^a^	0.216 ± 0.063 ^a^	1.445 ± 0.062 ^a^	1.433 ± 0.410 ^a^
	Summer, 2019	0.098 ± 0.041 ^b^	0.109 ± 0.026 ^b^	0.624 ± 0.242 ^b^	0.694 ± 0.137 ^b^
	Total	0.112 ± 0.068 ^B^	0.126 ± 0.067 ^B^	0.722 ± 0.440 ^B^	0.802 ± 0.440 ^B^
Mussel	Rainy, 2017	0.036 ± 0.029 ^c^	0.116 ± 0.135 ^a^	0.290 ± 0.225 ^c^	0.878 ± 0.876 ^a^
	Winter, 2018	0.095 ± 0.052 ^a^	0.101 ± 0.070 ^a^	1.669 ± 2.777 ^a^	1.039 ± 0.630 ^a^
	Summer, 2019	0.062 ± 0.039 ^b^	0.103 ± 0.073 ^a^	0.599 ± 0.382 ^b^	0.934 ± 0.631 ^a^
	Total	0.064 ± 0.047 ^C^	0.107 ± 0.098 ^B^	0.853 ± 1.728 ^B^	0.950 ± 0.725 ^B^
Cockle	Rainy, 2017	0.080 ± 0.040 ^c^	0.252 ± 0.077 ^b^	0.509 ± 0.227 ^c^	1.625 ± 0.551 ^b^
	Winter, 2018	0.214 ± 0.071 ^a^	0.523 ± 0.174 ^a^	1.438 ± 0.420 ^a^	3.803 ± 1.860 ^a^
	Summer, 2019	0.159 ± 0.063 ^b^	0.596 ± 0.229 ^a^	1.011 ± 0.332 ^b^	3.807 ± 1.127 ^a^
	Total	0.151 ± 0.081 ^A^	0.457 ± 0.227 ^A^	0.987 ± 0.505 ^A^	3.078 ± 1.654 ^A^

a, b, c: different superscript letters denote significant differences in heavy metals among the same species collected in different years (*p* < 0.05). A, B, C: different superscript letters denote significant differences in heavy metals among different species (*p* < 0.05).

**Table 2:** Numbers of microplastic (MP)-like particles in bivalve mollusks presented as mean ± SD (min–max).

Type of Bivalve	Collection Year	Number of MP-like Particles	
		Item/g Wet wt.	Item/Individual
Clam	Rainy, 2017	0.08 ± 0.10 ^b^ (ND-0.30)	0.18 ± 0.23 ^b^ (ND-0.68)
	Winter, 2018	0.13 ± 0.12 ^a^ (ND-0.30)	0.30 ± 0.28 ^a^ (ND-0.68)
	Summer, 2019	0.03 ± 0.10 ^c^ (ND-0.30)	0.08 ± 0.23 ^c^ (ND-0.68)
	Total	0.06 ± 0.04 ^B^ (ND-0.30)	0.14 ± 0.35 ^C^ (ND-0.68)
Mussel	Rainy, 2017	0.11 ± 0.17 ^a^ (ND-0.50)	0.95 ± 1.42 ^a^ (ND-4.26)
	Winter, 2018	0.03 ± 0.09 ^c^ (ND-0.30)	0.28 ± 0.80 ^c^ (ND-2.55)
	Summer, 2019	0.067 ± 0.11 ^b^ (ND-0.30)	0.57 ± 0.90 ^b^ (ND-2.55)
	Total	0.07 ± 0.13 ^B^ (ND-0.50)	0.60 ± 1.11 ^A^ (ND-4.26)
Cockle	Rainy, 2017	0.18 ± 0.20 ^a^ (ND-0.60)	0.55 ± 0.62 ^a^ (ND-1.87)
	Winter, 2018	0.04 ± 0.08 ^b^ (ND-0.20)	0.14 ± 0.26 ^b^ (ND-0.62)
	Summer, 2019	0.15 ± 0.40 ^a^ (ND-1.20)	0.47 ± 1.23 ^a^ (ND-3.73)
	Total	0.12 ± 0.26 ^A^ (ND-1.20)	0.38 ± 0.81 ^B^ (ND-3.73)

a, b, c: different superscript letters denote significant differences in MPs among the same species collected in different years (*p* < 0.05). A, B, C: different superscript letters denote significant differences in MPs among different species (*p* < 0.05).

**Table 3:** Exposure to Pb and margin of exposure (MOE) due to bivalve consumption by the Thai population.

Bivalve/ Age Group	Exposure to Pb (µg/kg bw/day)						MOE					
	3–5.9	6–12.9	13–17.9	18–34.9	35–64.9	≥65	3–5.9	6–12.9	13–17.9	18–34.9	35–64.9	≥65
Average exposure scenario (average consumption × average content)												
Clam	0.0018	0.0018	0.0011	0.0014	0.0012	0.0005	278	278	573	450	525	1260
Mussel	0.0036	0.0029	0.0016	0.0025	0.0013	0.0005	139	172	394	252	485	1260
Cockle	0.0138	0.0126	0.0083	0.0080	0.0039	0.0014	36	40	76	79	162	450
Total	0.0192	0.0173	0.011	0.0119	0.0064	0.0024	26	29	57	53	98	263
High-concentration exposure scenario (average consumption × 97.5 PCTL content)												
Clam	0.0042	0.0044	0.0027	0.0033	0.0027	0.0012	119	114	233	191	233	525
Mussel	0.0093	0.0075	0.0042	0.0063	0.0034	0.0013	54	67	150	100	185	485
Cockle	0.0263	0.0240	0.0159	0.0153	0.0075	0.0027	19	21	40	41	84	233
High consumer-exposure scenario (97.5 PCTL consumption × average content)												
Clam	0.0200	0.0207	0.0129	0.0153	0.0109	0.0058	25	24	49	41	58	109
Mussel	0.0492	0.0254	0.0159	0.0202	0.0133	0.0053	10	20	40	31	47	119
Cockle	0.0730	0.1510	0.0943	0.0798	0.0397	0.0113	7	3	7	8	16	56
Worst-case exposure scenario (97.5 PCTL consumption × 97.5 PCTL content)												
Clam	0.0477	0.0493	0.0308	0.0365	0.0259	0.0138	49	41	20	17	24	46
Mussel	0.1266	0.0654	0.0409	0.0519	0.0344	0.0137	37	29	15	12	18	46
Cockle	0.1395	0.2884	0.1802	0.1525	0.0758	0.0216	8	10	3	4	8	29

**Table 4:** Exposure to Cd and hazard quotient (HQ) due to bivalve consumption by the Thai population.

Bivalve/ Age Group	Exposure to Cd (µg/kg bw/month)						HQ of Exposure to Cd					
	3–5.9	6–12.9	13–17.9	18–34.9	35–64.9	≥65	3–5.9	6–12.9	13–17.9	18–34.9	35–64.9	≥65
Average exposure scenario (average consumption × average content)												
Clam	0.0597	0.0617	0.0385	0.0471	0.0386	0.0170	0.002	0.002	0.002	0.002	0.002	0.001
Mussel	0.1816	0.1464	0.0825	0.1228	0.0660	0.0257	0.007	0.006	0.003	0.005	0.003	0.001
Cockle	1.2483	1.1373	0.7563	0.7245	0.3564	0.1274	0.050	0.045	0.030	0.029	0.014	0.005
Total	1.4896	1.3454	0.8773	0.8944	0.461	0.1701	0.060	0.054	0.035	0.036	0.018	0.007
High-concentration exposure scenario (average consumption × 97.5 PCTL content)												
Clam	0.1221	0.1262	0.0788	0.0962	0.0790	0.0349	0.005	0.005	0.003	0.004	0.003	0.001
Mussel	0.5921	0.4771	0.2689	0.4004	0.2153	0.0838	0.024	0.019	0.011	0.016	0.009	0.003
Cockle	2.6333	2.3990	1.5955	1.5284	0.7519	0.2687	0.105	0.096	0.064	0.061	0.030	0.011
High consumer-exposure scenario (97.5 PCTL consumption × average content)												
Clam	0.6726	0.6952	0.4344	0.5144	0.3653	0.1938	0.027	0.028	0.017	0.021	0.015	0.008
Mussel	2.4626	1.2726	0.7952	1.0095	0.6687	0.2666	0.099	0.051	0.032	0.040	0.027	0.011
Cockle	6.6150	13.6805	8.5484	7.2347	3.5958	1.0230	0.265	0.547	0.342	0.289	0.144	0.041
Worst-case exposure scenario (97.5 PCTL consumption × 97.5 PCTL content)												
Clam	1.3758	1.4220	0.8885	1.0523	0.7471	0.3965	0.055	0.057	0.036	0.042	0.030	0.016
Mussel	8.0278	4.1486	2.5923	3.2909	2.1798	0.8691	0.321	0.166	0.104	0.132	0.087	0.035
Cockle	13.9540	28.8584	18.0324	15.2613	7.5851	2.1580	0.558	1.154	0.721	0.610	0.303	0.086

**Table 5:** Assessment of exposure to MP-like particles due to bivalve consumption by the Thai population.

Bivalve/ Age Group	Exposure to MP-like Particles Due to Bivalve Consumption (Items/Person/Day)					
	3–5.9	6–12.9	13–17.9	18–34.9	35–64.9	≥65
Average exposure scenario (average consumption × average content)						
Clam	0.016	0.033	0.033	0.047	0.039	0.015
Mussel	0.068	0.107	0.096	0.169	0.092	0.031
Cockle	0.188	0.332	0.353	0.400	0.198	0.062
Total	0.272	0.472	0.482	0.616	0.329	0.108
High-concentration exposure scenario (average consumption × 97.5 PCTL content)						
Clam	0.082	0.164	0.164	0.236	0.195	0.076
Mussel	0.360	0.562	0.507	0.892	0.483	0.165
Cockle	1.297	2.286	2.433	2.754	1.364	0.428
High consumer-exposure scenario (97.5 PCTL consumption × average content)						
Clam	0.185	0.369	0.369	0.517	0.369	0.172
Mussel	0.928	0.928	0.928	1.392	0.928	0.325
Cockle	0.997	3.991	3.991	3.991	1.996	0.499
Worst-case exposure scenario (97.5 PCTL consumption × 97.5 PCTL content)						
Clam	0.923	1.846	1.846	2.583	1.846	0.860
Mussel	4.888	4.888	4.888	7.332	4.888	1.711
Cockle	6.872	27.502	27.502	27.502	13.758	3.436

## Data Availability

The datasets generated to obtain the results presented in this article. are available from the corresponding authors upon reasonable request (pharrunrat.tan@mahidol.ac.th and w.karnpanit@westernsydney.edu.au).
